# Supplementary figures and images for: The Role of Alternative Splicing Factors, DDB2-Related Ageing and DNA Damage Repair in the Progression and Prognosis of Stomach Adenocarcinoma Patients
Source: Genes (Basel). 2022 Dec 23;14(1):39. doi: 10.3390/genes14010039 (PMC9858704; doi:10.3390/genes14010039)

Supplemental figure S1

a

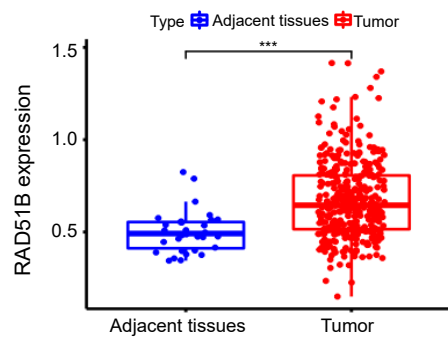

b

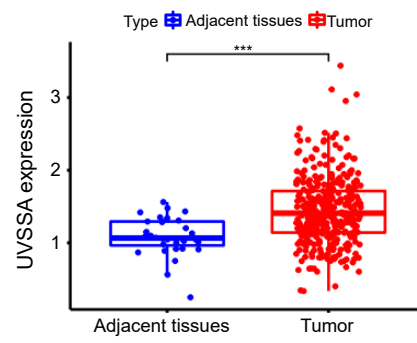

c

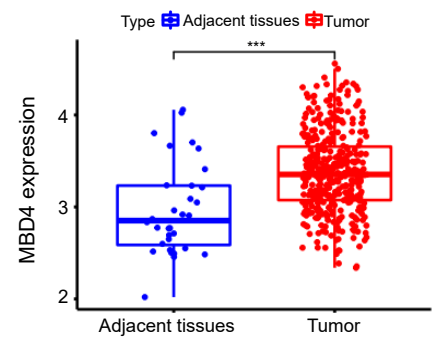

d

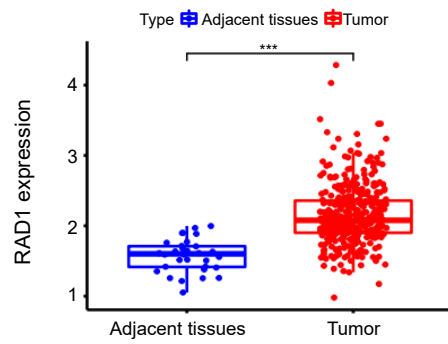

Supplement: Supplementary file 1 [file genes-14-00039-s001.zip › supplemental figure s1.pdf]

Supplemental figure S2

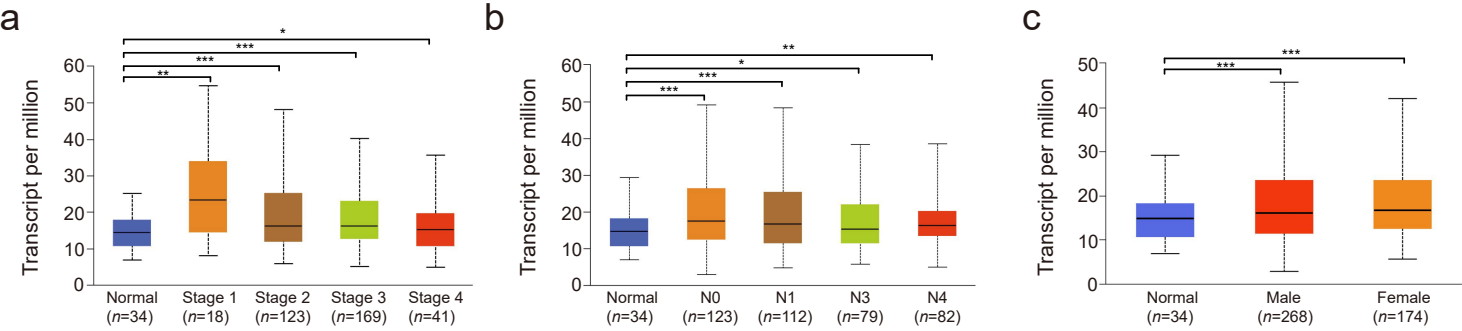

Supplement: Supplementary file 1 [file genes-14-00039-s001.zip › supplemental figure s2.pdf]

Supplemental figure S3

a

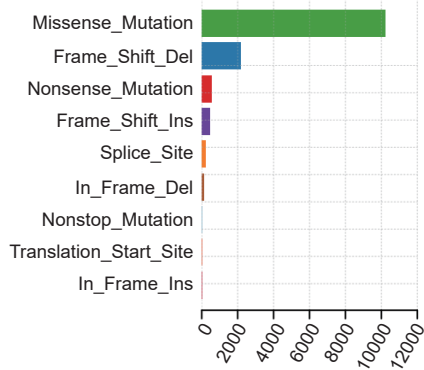

b

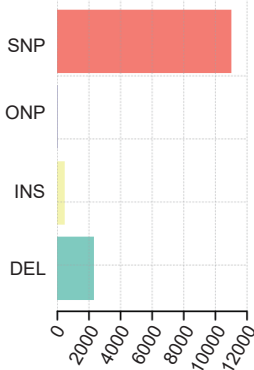

c

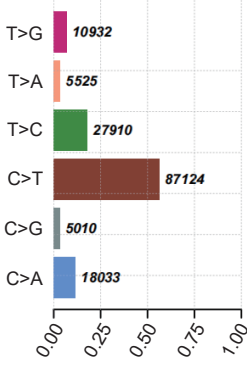

Supplement: Supplementary file 1 [file genes-14-00039-s001.zip › Supplemental figure s3.pdf]
